# Strategies for Achieving High Sequencing Accuracy for Low Diversity Samples and Avoiding Sample Bleeding Using Illumina Platform

**DOI:** 10.1371/journal.pone.0120520

**Published:** 2015-04-10

**Authors:** Abhishek Mitra, Magdalena Skrzypczak, Krzysztof Ginalski, Maga Rowicka

**Affiliations:** 1 Department of Biochemistry and Molecular Biology, University of Texas Medical Branch at Galveston, 301 University Blvd, Galveston, TX, 77555, USA; 2 Institute for Translational Sciences, University of Texas Medical Branch at Galveston, 301 University Blvd, Galveston, TX, 77555, USA; 3 Laboratory of Bioinformatics and Systems Biology, Centre of New Technologies, University of Warsaw, Zwirki i Wigury 93, 02-089 Warsaw, Poland; 4 Sealy Center for Molecular Medicine, University of Texas Medical Branch at Galveston, 301 University Blvd, Galveston, TX, 77555, USA; VU University Medical Center, NETHERLANDS

## Abstract

Sequencing microRNA, reduced representation sequencing, Hi-C technology and any method requiring the use of in-house barcodes result in sequencing libraries with low initial sequence diversity. Sequencing such data on the Illumina platform typically produces low quality data due to the limitations of the Illumina cluster calling algorithm. Moreover, even in the case of diverse samples, these limitations are causing substantial inaccuracies in multiplexed sample assignment (sample bleeding). Such inaccuracies are unacceptable in clinical applications, and in some other fields (e.g. detection of rare variants). Here, we discuss how both problems with quality of low-diversity samples and sample bleeding are caused by incorrect detection of clusters on the flowcell during initial sequencing cycles. We propose simple software modifications (Long Template Protocol) that overcome this problem. We present experimental results showing that our Long Template Protocol remarkably increases data quality for low diversity samples, as compared with the standard analysis protocol; it also substantially reduces sample bleeding for all samples. For comprehensiveness, we also discuss and compare experimental results from alternative approaches to sequencing low diversity samples. First, we discuss how the low diversity problem, if caused by barcodes, can be avoided altogether at the barcode design stage. Second and third, we present modified guidelines, which are more stringent than the manufacturer’s, for mixing low diversity samples with diverse samples and lowering cluster density, which in our experience consistently produces high quality data from low diversity samples. Fourth and fifth, we present rescue strategies that can be applied when sequencing results in low quality data and when there is no more biological material available. In such cases, we propose that the flowcell be re-hybridized and sequenced again using our Long Template Protocol. Alternatively, we discuss how analysis can be repeated from saved sequencing images using the Long Template Protocol to increase accuracy.

## Introduction

Next generation sequencing technology is rapidly developing and has become one of the most popular and crucial techniques used today to answer key biomedical questions. The currently dominant sequencing platform is Illumina, used for ∼85% of samples deposited in NCBI’s Sequence Read Archive (SRA) in 2013 [1]. Many important applications of next generation sequencing (e.g. reduced representation sequencing [2], microRNA sequencing [3], Hi-C technologies [4] and any technique employing custom barcodes, e.g. [5]) result in sequencing libraries with low sequence diversity in the initial bases of the sequenced reads.

The standard Illumina data analysis protocol uses only images corresponding to the first four positions in the reads to determine the coordinates of different clusters on the flowcell, which is a key step in sequencing image analysis. Therefore, sequencing libraries with low sequence diversity in the initial four positions leads to sequencing images that pose a considerable challenge to the image recognition algorithm and usually results in low quality data when using the Illumina platform. Moreover, the same software issue that lowers quality of data originating from low initial sequence diversity samples is also a major source of sequencing errors in normal samples.

Since the software design creates the problem, we maintain that the most appropriate and logical way to correct it is by modifying the software itself. Therefore, we developed an approach—Long Template Protocol—that solves this problem for the most popular Illumina HiSeq 2000 and HiSeq 2500 platforms. A computational solution to rectify this problem was proposed for the previous Illumina sequencer, Genome Analyzer II [6, 7]. Unfortunately, for reasons discussed below, these methods are not feasible while sequencing on HiSeq. Our solution is to use images corresponding to more than the first four nucleotides to distinguish between clusters of nearby reads on the flowcell. This approach not only very substantially increases the quality of data originating from low initial sequence diversity sample but also improves sequencing accuracy of normal diversity samples and reduces “sample bleeding” (incorrect assignment of the multiplexed samples), and is thus of general interest.

For comprehensiveness, we not only present our software modification strategy to improve quality of the Illumina sequencing data (Long Template Protocol), but we also discuss preventive strategies that can sometimes be implemented at the experiment design stage (Approaches 1 – 3), as well as how to rescue results of an unsuccessful sequencing when repeating the sequencing is not an option, e.g. due to scarcity or cost of a sample (Rescue strategies, Approach 4 & 5).

### Sequencing technology

**Fig 1:**
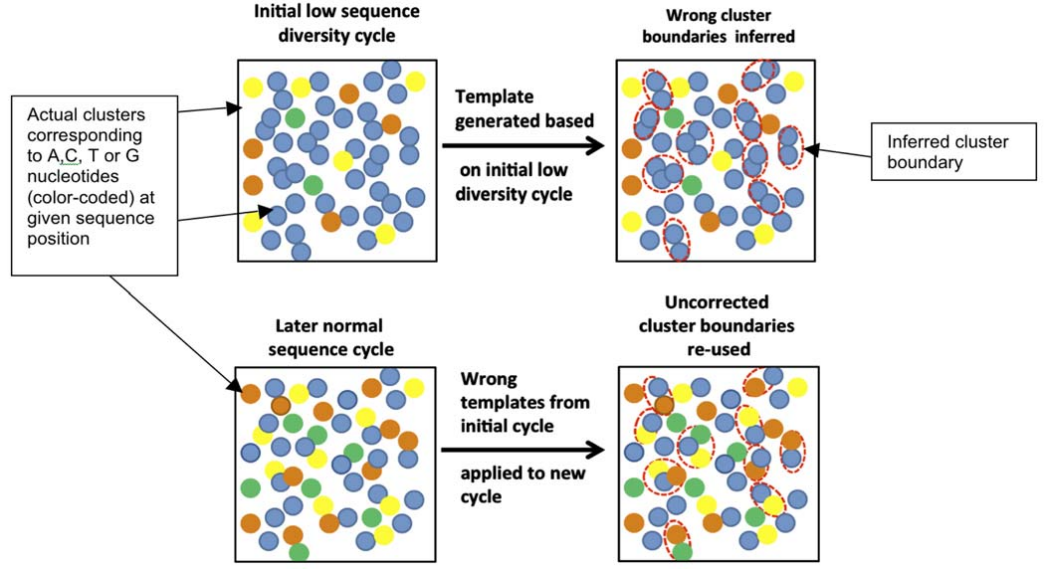
Example of differences between base frequencies and raw images from sequencing a low initial sequence diversity sample and a diverse sample. The low initial sequence diversity sample is highlighted in red (top panel) and a diverse sample is highlighted in blue (bottom panel). Per cycle intensity (which correlates with base frequency) pie charts are shown at the top for each sample. The low diversity sample (top) has an 11bp 5′ -barcode, that causes different base frequencies than in the diverse human sample (bottom). For each sample, the bottom panel shows raw sequencing images from the first cycle of sequencing. The imbalanced frequency distribution in the top panel (barcoded, low diversity sample, highlighted in red) is due to over-representation of the base ‘T’, the first base in the barcode. There is also some signal from base ‘C’ due to linker sequencing.

HiSeq builds upon the sequencing by synthesis method utilized in the Illumina Genome Analyzer. In this method, fragmented DNA is first attached to the top and the bottom surfaces of a glass flowcell using specific oligonucleotide adapters. DNA is then amplified using the procedure called bridge amplification, which is based on the polymerase chain reaction (PCR). During bridge amplification *clusters* of identical DNA fragments are generated on the flowcell. Later, they undergo the following sequencing by synthesis *cycles*: a single complementary base for each strand of DNA on the flowcell is incorporated, using a fluorescently labeled deoxynucleotide (dNTP). After incorporating complementary, fluorescently labeled bases, the flowcell undergoes imaging. Lasers of two separate wavelengths excite the fluorophores while a camera captures images of the flowcell. Each flowcell consists of several vertical lanes and each lane is divided into two or three logical vertical partitions, called swaths. The camera moves vertically across the swaths and obtains four images, each one corresponding to different nucleotide (Fig 1). Both the bottom and the top surfaces of each lane of the flowcell are independently imaged. Thereafter, the fluorescent dye is removed and then the next corresponding dNTP is incorporated and imaged, beginning the cycle anew. Incorporated dNTPs are identified on the images as lighted spots.

### Sequencing Image Analysis

Determining the location of each cluster on the flowcell in relation to the image coordinates (*template generation*), is performed during the first four sequencing cycles and is used to register images from subsequent sequencing cycles. Specifically, to estimate positions of clusters on the flowcell, the current version of the HiSeq Control Software (HCS) v1.5 [8] performs the following procedure:

- Images from the first four sequencing cycles (corresponding to the first four bases of the sequenced read) are examined and positions of the clusters are determined in each cycle separately. Number of clusters per cycle is computed.
- Cycle with the highest number of clusters is called a golden cycle, second best is called silver.
- Positions of the clusters are estimated based on the golden cycle and the silver cycle, thus creating a *template*.
- Images from any other cycle are aligned against the template by finding cycle-specific offset.

After image analysis steps, the appropriate bases are called and finally reported as sequences in text format for further analysis.

### Why lack of sequence diversity in the initial positions causes problems

Fast speed of template generation is highly desirable in the current Illumina setup, where it is performed during the sequencing run. To increase speed and reduce computational cost, in the standard Illumina protocol only images from the first four sequencing cycles are used for generating templates (which are used later for cluster calling). The first four cycles are selected for template generation because of the implicit assumption that they are most likely to be of the highest quality. However, when a sample lacks initial sequence diversity, then cluster recognition is actually most difficult in the initial cycles, which is why the above algorithm creates problems with overall data quality in Illumina sequencing of low initial sequence diversity samples [9].

**Fig 2:**
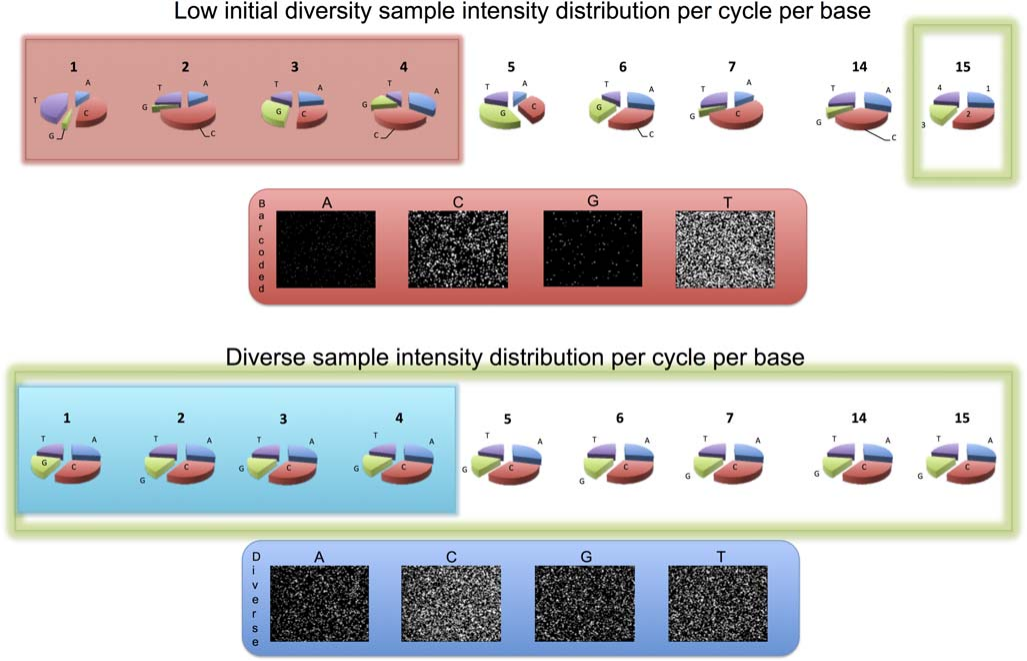
Lack of sequence diversity in first four positions causes errors in base calling in later cycles. Template used for cluster calling is created using only data from the first four cycles (top). Once the template is generated, it is never corrected (bottom). Templates created based on low-sequence diversity cycles tend to be of poor quality (bottom right) and result in poor cluster detection for later, normal diversity cycles as well, thus globally compromising sequencing quality.

The top panel of Fig 1 shows HiSeq images from sequencing of our low initial sequence diversity test samples (BLESS data). In the BLESS data [5], the majority of reads begin with a ‘TCGAG’ 5′-barcode, thus creating low initial sequence diversity. Examination of the top panel of Fig 1 shows that in the first sequencing cycle, the majority of the signal is observed in the channel corresponding to ‘T’ nucleotide, which is first in the BLESS ‘TCGAG’ barcode (there is also visible signal in the ‘C’ channel, which originates from the linkers used). The bottom panel of Fig 1 shows analogous raw images and intensity pie charts for a sequence-diverse sample. The BLESS data exhibit characteristic properties of the low initial sequence diversity data: the signal spots in one of the channels become more dense than normally observed, thus making it very difficult to accurately detect cluster boundaries (Fig 1, top panel, channel ‘T’). A typical error then is mistakenly identifying two or more clusters as one because, in the low-diversity cycle, they share the same nucleotide at the examined position (Fig 2, top right). Fig 2 also shows (bottom panel) that template generation performed at later cycles, is much better for low initial sequence diversity samples. Unfortunately, in the standard approach, cluster boundaries, once derived, are used for later cycles without corrections (Fig 2, bottom panel). Therefore, mistakes in detecting cluster boundaries during cluster generation, propagate to later, “normal” cycles resulting in detection of undesirable mixed clusters, with high intensities from at least two different bases [6], as illustrated in Fig 2, bottom right. Such clusters are likely to be rejected later during image analysis by the purity filter. Thus low initial sequence diversity adversely affects not only quality of the base calling in the region of low sequence diversity, but also along the entire length of the sequenced read. This problem can be somewhat mitigated by lowering cluster density of the sample that is loaded onto the flowcell (Approach 3), because it is easier to detect cluster boundaries if overall cluster density is lower (Fig 2).

### Sample bleeding

Another quality problem related to inaccuracies of the standard Illumina protocol in detecting cluster boundaries is sample bleeding. Sample bleeding is the incorrect assignment of reads to multiplexed samples that are being sequenced in the same sequencing lane. Until very recently, it has been thought that the main source of sample bleeding not related to the cross-contamination during library preparation, is incorrect multiplexing index sequencing, leading to erroneous assignments of reads to samples. Therefore, effort was made to design barcodes that are unlikely to be changed into another through sequencing errors, with the gold standard being that at least 3 sequencing errors would have to occur simultaneously to morph one index into another (corresponding to the Hamming distance of at least 3). In a recent seminal paper [10], Kircher and colleagues showed that reads that are assigned to the incorrect sample tend to not have a multiplexing index similar to the mis-assigned sample, but rather are located on the flowcell in close proximity to the cluster of reads to which they are later mis-assigned, pointing towards problems with cluster detection, not with multiplexing index similarity, as the most likely cause of sample bleeding. However, library preparation of Kircher and colleagues was done in a manner that did not exclude the possibility that sample contamination may occur during library preparation. Therefore, we repeated their experiments preparing samples in a way that excludes possibility of cross-contamination during library preparation and researched if improving cluster detection through our Long Template Protocol would reduce sample bleeding. Our results reported in this paper further support conclusions of Kircher and colleagues [10].

## Results

As we explained in the introduction, the problem with sequencing low diversity data on the Illumina platform is caused by the fixed template based on first four sequencing cycles that does not take into account possible low diversity sequence at the beginning of the read. There is nothing in the sequencing technology that would cause samples with low-diversity reads at the beginning to be more difficult to sequence than reads with the analogous low diversity region in the middle of the read. The problem is solely created by the Illumina analysis software. Therefore, we contend that the simplest and most logical solution is to correct the problem at the software level. Due to the non-open source nature of the Illumina software, not all modifications are possible, but we have developed several simple-to-implement modifications (Long Template Protocol) that have strong positive impact on the quality of the sequencing of low diversity data on the Illumina platforms. The Long Template Protocol also increases quality of the sequencing data for normal diversity samples and reduce sample bleeding, although these effects are not of such magnitude as for low-diversity data. The technical details of the Long Template Protocol are described in the Materials and Methods. Here, we present the results showing improved data quality versus the standard Illumina protocol. For comprehensiveness, we also discuss five alternative approaches that can be used to solve the quality problems with sequencing low-diversity data. We also include comparison and analysis of all discussed approaches with respect to time and costs needed, as well as their benefits and limitations.

### Long Template Protocol

The most logical and straightforward solution to the problem of accurate template generation with low initial sequence diversity is to defer template generation for a user defined number of initial cycles, thus avoiding the initial low-sequence diversity region (e.g. barcode). Unfortunately, the current version of HCS (HiSeq Control Software) has likely deprecated the use of such a switch, “FirstTemplateCycle”, present in the version of this software used for Genome Analyzer II (we tested this by adding this option manually to the configuration file with a value higher than 1, it did not result in delayed template generation). The only remaining possibility to achieve templates based not only on a non-diverse initial region is thus to extend templates beyond the non-diverse region. In HCS, there is an option, “TemplateCycleCount”, with a default value of 4. This option implements the template generation from the first four cycles of images, starting from the cycle 1. Thus, by modifying the value of “TemplateCycleCount” parameter in the appropriate configuration files, one can achieve generation of longer templates during sequencing (described in detail in Materials and Methods).

We have also observed that for each sequencing cycle on the v1.5 flowcell, HiSeq generates 16 (4 (bases) x 2 (surfaces) x 2 (swaths)) 16-bit TIF images of size 2048 x 160000 pixels. The theoretical memory requirement for loading 4 images from each cycle per surface per swath is roughly 2.6GB. HCS loads 4 cycles worth of image data simultaneously in the memory to calculate the template and thus the memory requirement per surface per swath for 4 cycles is approximately 10.4GB. With an increased number of cycles used for template generation, the memory requirements would theoretically scale linearly and that is what the Illumina software manual states [8]. Thus the memory requirement for loading data from 20 cycles would be 52GB, which slightly exceeds the default memory capacity (48GB) of the *Dell T7500* workstation running HCS. We therefore speculate that HCS limits the template length to 4 to avoid exceeding available RAM memory.

To estimate memory usage for our Long Template Protocol, we tested how the memory usage scales with the number of cycles used for template generation. We generated templates of length 20 with peak memory requirements during template generation of about 80GB, as determined by monitoring the ‘Memory Commit Size’ in Windows Task Manager. This exceeded allowable size of 48GB of the Dell T7500 workstation memory. Since it was not possible to revert the HCS software to generate shorter templates at that point, we calculated templates by reducing the number of execution threads from 8 to 1, thus obtaining data from approximately 1/8th of the flowcell. We repeated this experiment using newer flowcell v3 and generating templates of length 8 and monitored memory usage continuously using Windows Task Manager. The maximal observed Memory Commit Size was about 32GB. That experiment shows that memory usage for template generation scales approximately linearly with the number of cycles used, and that the approximate formula for the maximal memory usage is about 4GB times length of the template, irrespective of the type of flowcell used (v1.5 or v3). This means that with the current setup the maximal length of templates allowable would be 12 for normal cluster density and proportionally longer for lower cluster density. These results also underscore the fact that experimentation with longer template generations must be undertaken carefully. A good permanent solution would be to upgrade the memory of the RTA (Real Time Analysis) computer; the standard configuration can be upgraded up to 192GB, which would allow template generation of almost arbitrary length, theoretically up to 48, if the scaling of memory requirements remains linear for such template lengths.

If the set value of “TemplateCycleCount” is high enough to cause exceeding available RAM memory during template generation and thus slows down the completion of sequencing in an acceptable time, the possible emergency solutions are:

(i) stop the HCS and restart it with reduced number of threads (through decreasing NumAnalysisThreads parameter).
(ii) stop the sequencing, re-hybridize the flowcell and start sequencing again using the same flowcell, but setting TemplateCycleCount option to lower value.

Necessary modifications are described in detail in Materials and Methods.

**Fig 3:**
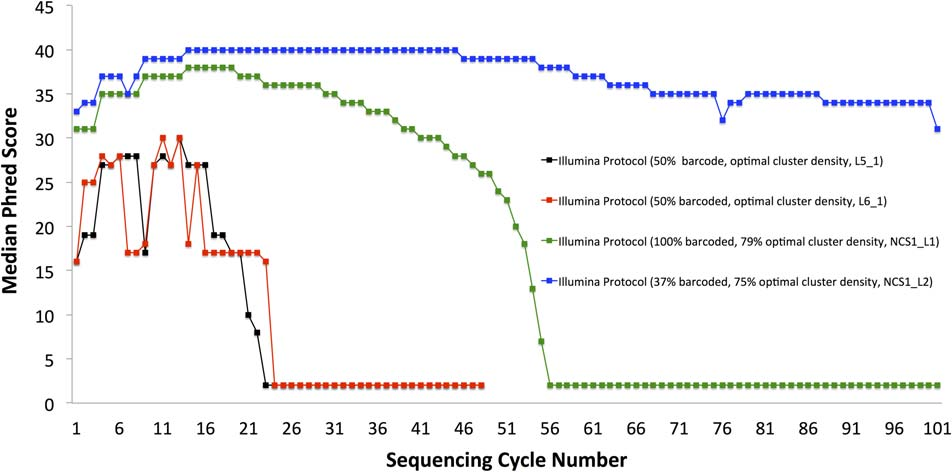
Long Template Protocol improves strikingly data quality, even for 100% non-diverse samples sequenced with normal cluster density. Median Phred quality scores per-base are shown. Same biological samples were sequenced using either the standard Illumina data analysis protocol and their recommended 50% spike-in with PhiX (black and purple) or using our Long Template Protocol with either normal cluster density and 90% spike-in (green) or as 100% non-diverse sample, either at normal cluster density (blue) or at lowered cluster density (red). Long Template Protocol sequencing results show invariably very high quality data, independent of the usage of spike-ins or lowered cluster density. Templates generated based on the first 20 cycles were used (non-diverse data included 11bp 5′-barcode).

**Fig 4:**
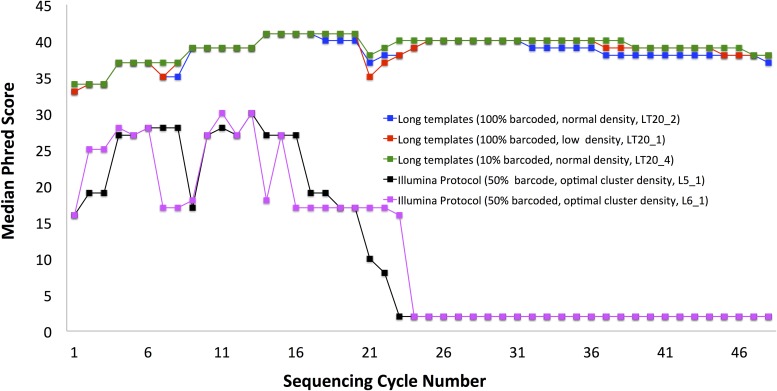
Severe dilution combined with lowering cluster density substantially improves data quality from sequencing low diversity samples. Shown are median Phred quality scores per-base of our low initial sequence diversity sample (referenced later as A4) (red and black), spiked in with 50% PhiX, the maximal dilution recommended by the manufacturer. Extremely low quality scores lead to a practically unusable sample. Also shown are improved scores of this same sample re-sequenced using more dilution and lowering cluster density, as we recommend (blue). Lowering cluster density alone does not improve the results if standard Illumina data analysis protocol is used (green).

The quality of the data obtained from usage of templates based on the first 20 cycles was remarkable, showing up to 70-fold increase in the number of correctly sequenced reads with the BLESS barcode, as compared with earlier sequencing of the same sample (Table 1). Most impressively, the partial data that we obtained from 1/8 of tiles (samples LT20_1, LT20_2 and LT20_4, rows 7-9, Table 1) contains a nominally higher number of our barcoded reads that are mapable to the genome than collectively from all our previous data sequenced following standard Illumina protocols for low sequence diversity data from 6 full lanes of HiSeq (rows 1-6, Table 1). Phred scores remained uniformly high for samples sequenced following the Long Template Protocol (Fig 3, showing samples LT20_1, LT20_2 and LT20_4) unlike for the sample that was sequenced using the default Illumina protocol (Fig 4). The results presented here demonstrate that it is possible to obtain high-quality data while sequencing low diversity samples, even under the most challenging conditions of normal cluster density (sample LT20_2 in Fig 4).

**Table 1:** Comparison of quality of sequencing of the BLESS samples using Illumina default protocol (template of length 4) and our Long Template Protocol (template of length 20).

F								
Sample	Protocol	% BLESS	% Control	Cluster density	# Reads	>Q30	% Barcoded	# Barcoded & mapped
A4	Illumina	100%	0%	100%	81,690,716	39%	1.1%	386,703
C3	Illumina	100%	0%	100%	93,541,636	36%	1.8%	1,095,261
L5_1	Illumina	50%	50%	67%	44,752,944	29%	1.0%	107,514
L5_2	Illumina	50%	50%	67%	44,752,944	63%	5.6%	704,185
L6_1	Illumina	50%	50%	67%	56,647,313	32%	3.0%	573,393
L6_2	Illumina	50%	50%	67%	56,647,313	61%	9.6%	1,598,766
LT20_1	Our (LTP)	100%	0%	60%	5,094,823	86%	**77.0%**	2,766,509
LT20_2	Our (LTP)	100%	0%	100%	7,827,274	84%	**77.0%**	4,100,771
LT20_4	Our (LTP)	10%	90%	75%	6,545,127	94%	**80.0%**	428,208

Columns: Sample name, template generation protocol used, % of low diversity sample, % of diverse control, cluster density on the flowcell (expressed as percentage of optimal cluster density), number and percentage of reads with Phred score >30, percentage of reads with intact BLESS barcode among reads with bases with Phred scores >30 normalized for the percentage of the BLESS sample (i.e. if 10% of the sample is BLESS and 10% of reads among Q30 reads are barcoded, that percentage would be normalized to 100%), number of reads with intact BLESS barcode mapped to the human genome with 0 errors. Note that samples A4, LT20_1 and LT20_2 are technical replicates and there is 70-fold increase in number of reads with intact BLESS barcode after switching to our Long Template Protocol.

**Fig 5:**
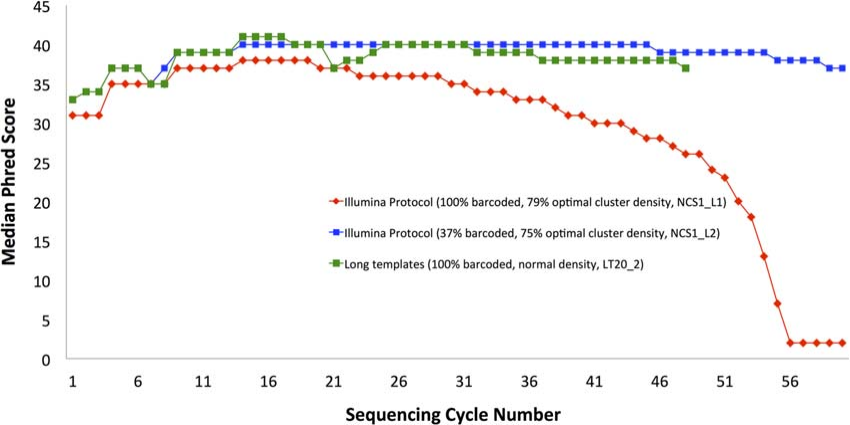
Diluting low-diversity sample and lowering cluster density results in quality improvements similar to Long Template Protocol, but substantially lowers sequencing yield. Median Phred scores of the bases called for each sequencing cycle are plotted. Blue plot shows the results of diluting low-diversity sample and lowering cluster density and sequencing using standard protocol (blue), while green plot illustrates the results of Long Template Protocol sequencing for 100% non-diverse sample at normal cluster density (green). For comparison, unsatisfactory results of sequencing non-diverse sample with 21% spike-in using standard Illumina data analysis protocol are shown (red).

To overcome this issue, we systematically tested impact of percentages of non-diverse samples on quality of the sequencing results. High cluster density can pose additional problems in cluster calling (Fig 1). Conversely, problems with cluster calling due to low sequence diversity of the sample can be, to some extent, mitigated by lowering the cluster density, allowing clusters to be located farther away from each other on the flow cell (Fig 1). We tested both the effects of the percentage of non-diverse sample and cluster density on the sequenced data quality. Results are shown in Fig 4 and Fig 5.

### Long Template Protocol reduces sample bleeding

It has been previously believed that major sources of sample bleeding (i.e. incorrect sample assignment in multiplexed sequencing runs) are errors in the multiplexing indices due to sequencing errors, library amplification or oligonucleotide synthesis. As a consequence, efforts to avoid sample bleeding were focused on designing indices with high Hamming distance (number of nucleotide substitutions required to turn one valid index into another). It is typically assumed that such errors should not occur with a frequency higher than 0.5% at any given position (0.5% failure rate corresponds to a Phred score of 23). As a consequence, if any indices with Hamming distance of at least 3 are used [11–14] as in the Kircher et al. study [10], errors in sample assignment theoretically (binomial model) should not be observed in more than 4.3 ⋅ 10^−6^ cases, while in practice they were observed in 3 ⋅ 10^−3^ cases, three orders of magnitude more often than expected. Assuming average Phred score of 30, chance of misreading a 6nt long Illumina multiplexing index among a set of indices with pairwise Hamming distance of at least 3 is even lower, < 2 ⋅ 10^−8^, and for average Phred score of 40 it is < 2 ⋅ 10^−11^. This means for good quality of base calling (Phred scores in 30s or 40s), sample bleeding caused by sequencing error within index should be practically unobservable or limited to only several reads per lane. In the Kircher et al. study [10], however, the possibility of sample cross-contamination during library preparation was not excluded. Therefore, we researched sample bleeding again, this time preparing libraries in a manner eliminating any possibility of sample cross-contamination. Indeed, we confirmed that in non-multiplexed sequencing lanes, only several reads from the sequenced human genome mapped to the yeast genome, as expected by chance (Table 2). Based on binomial probability distribution, since we used Illumina TruSeq indices with Hamming distance of at least 4, probability of one index randomly morphing into another valid one is only 9.3 ⋅ 10^−9^. In contrast with this low estimate, in samples multiplexed with the Illumina TruSeq kit, we observed sample bleeding with the frequency of 3.5 ⋅ 10^−4^ for the very good quality data and up to almost 20% for very low quality data (Table 2). Motivated by Kircher et al., who showed that the cause of sample bleeding is incorrect cluster calling, not errors in index sequencing, we researched if our Long Template Protocol reduces sample bleeding. As Table 2 demonstrates, Long Template Protocol reduces the frequency of sample bleeding in all samples examined, although the problem is still present. Therefore, if experiments with multiplexed samples are planned and high accuracy is desired, we recommend employing Long Template Protocol and to only multiplex for sequencing in the same lane samples from evolutionary distant organisms. In such case, the Long Template Protocol will substantially reduce sample bleeding, and remaining sample bleeding will usually be easy to identify for evolutionary distant organisms. If evolutionary distant samples are not available for multiplexing, cluster density should also be lowered and longer templates (as long as feasible) may be used, in order to maximize sequencing accuracy. Another possibility is to use additional indices, as recommended by Kircher et al., although this is in our opinion a more costly and laborious solution.

**Table 2:** Our Long Template Protocol reduces sample bleeding by accurate index assignments.

% Yeast	Protocol	Multiplexed	Obs. sample bleeding	Exp. sample bleeding	Obs/Exp ratio
61%	Illumina	Yes	3.5E-04	8.5E-07	412
20%	Our	Yes	7.3E-05	8.5E-07	86
40%	Our	Yes	1.2E-04	8.5E-07	141
60%	Our	Yes	1.5E-04	8.5E-07	176
80%	Our	Yes	1.9E-04	8.5E-07	224
90%	Our	Yes	1.7E-04	8.5E-07	200
0%	Our	No	8.1E-07	8.5E-07	0.96
0%	Our	No	8.9E-07	8.5E-07	1.05
% PhiX	Protocol	Multiplexed	Obs. sample bleeding	Exp. sample bleeding	Obs/Exp ratio
50%	Illumina	Yes	1.5E-01	1.6E-07	937500
50%	Illumina	Yes	2.0E-01	1.6E-07	1250000
0%	Our	No	0(< 2.0E-07)	1.6E-07	0
0%	Our	No	0(< 1.3E-07)	1.6E-07	0

Comparison of sample bleeding in multiplexed samples, sequenced with either standard Illumina protocol (“Illumina”) or our Long Template Protocol (“Our”). Columns: Percentage of non-human sample (top table shows results for yeast, bottom table for PhiX), template protocol, multiplexed (yes or no), observed sample bleeding, expected sample bleeding, observed to expected ratio. Sample bleeding was estimated as a percentage of reads in the human sample that do not map to the human genome, but map without mismatches to the control organism genome (see Materials and Methods, in the last two rows of each samples that are 100% human estimated sample bleeding is the observed false positive frequency for our test). Note that sample bleeding is the highest for Illumina Standard Protocol and that Long Template Protocol can reduce sample bleeding by 2-5 times as compared to Standard Illumina Protocol, although still remains substantial.

### Alternative approaches

We also compared several other approaches to achieving high-quality data from low sequence diversity samples. To this end, we performed the following experiments:

- mixing low diversity samples with high-diversity samples (Approach 2)
- lowering cluster density (Approach 3)
- flowcell rehybridization (Approach 4) followed by sequencing using the Long Template Protocol (rescue strategy)
- offline image analysis from previously saved images (Approach 5)

For comprehensiveness, we also include discussion of how low sequence diversity can be avoided, if it is caused by usage of in-house barcodes (Approach 1). We discuss these alternative approaches in chronological order, that is we start with strategies that can be only implemented before samples are prepared and end with rescue strategies for important samples, in cases when sequencing yielded unsatisfactory quality data due to low diversity (or sample bleeding) and no more biological material is available.

### Approach 1. Experimental design: Avoid low initial sequence diversity by (re-)designing diverse barcodes

Before the samples are derived, it is possible to avoid the aforementioned problem by ensuring sequence diversity in the initial bases. In many sequencing applications, this would require the re-design of traditional barcodes or introducing additional, sequence diverse barcodes in front of those traditionally used. For example, the original BLESS protocol [5] relies on the 5′-end, in-house barcode ‘TCGAG’ that results in low initial sequence diversity samples. To avoid this problem, new BLESS barcodes can be redesigned to not cause low initial sequence diversity. To this end, instead of the restriction enzyme XhoI, used in the original BLESS protocol, may be replaced with AasI, which has the following recognition site:

5′…G A C N N N N ^^^N N G T C…3′
3′…C T G N N ^^^N N N N C A G…5′.

where N denotes an arbitrary nucleotide. Thus, the sequence ‘TCGAG’ used in original BLESS barcodes [5] will be replaced in synthesized primers with the following four sequences:

GAC**GT**GC**AC**GTC; (resulting BLESS barcode: **AC**GTC),
GAC**AG**GC**CT**GTC; (resulting BLESS barcode: **CT**GTC),
GAC**CA**GC**TG**GTC; (resulting BLESS barcode: **TG**GTC),
GAC**TC**GC**GA**GTC; (resulting BLESS barcode: **GA**GTC).

Note that all of these can be cut with the same restriction enzyme, AasI, but would result in four different BLESS barcodes (above, variable part highlighted in bold), thus increasing sequence diversity. This approach can only be used before sequencing libraries are prepared. *However, we stress that there is nothing inherent in sequencing technology that is incompatible with 5′ barcoding*; the problem is created purely at the level of the sequencer’s software. Therefore, rather than redesign entire experimental protocols, and test and optimize new primers and restriction enzymes, it is rather recommended to rectify the problem by using our Long Template Protocol.

### Approach 2 & 3. After library preparation: Lowering cluster density and/or mixing with diverse sample

Once the libraries are prepared, it is impossible to modify 5′-ends of the reads to be sequenced, although as noted it is possible to lower the density of same bases (e.g. ‘T’, blue dots in Fig 2) by lowering the overall cluster density on the flowcell through mixing a diverse sample with a low-diversity sample. Mixing with another non-diverse sample, containing mostly complementary nucleotides at the shared low-diversity region would also solve the problem, although samples satisfying these complex criteria are much harder to find than samples that are just diverse, hence we will omit this option from further discussion. Illumina recommends mixing the low diversity sample with up to 50% of PhiX (sequencing control) [15]. In our experience, to obtain high-quality data from low diversity sample, often dilution with more than 50% of PhiX is required, especially if cluster density is not lowered (Fig 4 and top rows of Table 1).

We conclude that by carefully controlling sample composition, as well as cluster density, one can successfully sequence low-diversity samples. The data indicates that using no more than 30-40% of low diversity sample, in combination with 65-75% of the optimal cluster density for a given flowcell is the best combination (Table 3 and blue plot in Fig 3) and yields results comparable to using Long Templates Protocol without diluting the low-diversity sample (Fig 5 and Table 4). However, using our Long Template Protocol, allows more efficient utilization of the sequencer by enabling higher cluster density or less spike-in control, without adverse effects on the data quality. Even with completely diverse sample, exceeding optimal cluster density (as given by flowcell manufacturer) is very detrimental for data quality. In case of low diversity data, this effect is even stronger (Fig 1 and Table 3) and no more than 65-75% of the optimal cluster density should be used.

**Table 3:** Results of experiments with adjusted cluster densities and control sample percentages (standard template).

Lane	% Low-diversity	Cluster density	No. of Reads	>Q30	% Barcoded	% Barcoded and Mapped
NCS1_L1	100%	100%	87,620,453	40%	72%	78%
NCS1_L2	37%	100%	53,788,830	87%	70%	96%

Columns: Lane, % of low diversity sample, cluster density (as in Table 1), total number of sample reads sequenced, percentage of base with Phred score >30, percentage of reads with intact barcode, percentage barcoded reads that mapped to genome.

**Table 4:** Results of experiments with rehybridized flowcell and Long Template Protocol (template of length 16).

Lane	Cluster density	Tiles	No. of Reads	>Q30	% Barcoded	% Barcoded and Mapped
LT16_1	40%	100%	42,702,989	93%	**79%**	97%
LT16_2	66%	100%	76,729,709	90%	**79%**	96%
LT16_4	60%	100%	62,927,691	94%	**80%**	96%

Columns: Lane, cluster density (as in Table 1), tiles processed successfully, total number of reads sequenced, percentage of bases with Phred score >30 (according to Illumina >85% indicates good quality data), normalized percentage of reads with intact barcode (as in Table 1), percentage barcoded reads that mapped to genome.

### Approach 4. After an unsuccessful sequencing run: rehybridization of the flowcell and re-sequencing using Long Template Protocol

We also tested usage of the Long Template Protocol as a rescue strategy, in the event that the initial sequencing run was unsuccessful and no additional biological material is available. To this end, we rehybridized the flowcell that was used in the experiments with templates of length 20. The rehybridization automatically reduces cluster density; in this study the reduced densities were 256, 395 and 390 K/mm^2^, respectively, for lanes 1, 2 and 4, which corresponded to 40%, 66% and 60% of their optimal cluster density. This further lowered cluster density gave no additional gains in data quality (compare Table 1 and Table 4), showing that there is no added benefit to lowering cluster density below 75% of the optimal when using Long Template Protocol. Most importantly, we showed that failure in the first attempt to sequence low-diversity samples can be solved by re-hybridization and re-sequencing using Long Template Protocol (Table 4). Quality of the data is the same as it was for cases in which the Long Templates Protocol was used during initial sequencing (Fig 5), except that the total number of sequenced reads is lowered due to rehybridization.

### Approach 5. Offline image analysis

Performing a sequencing run with the default 4-first cycle templates, while storing images as a backup to perform offline longer template analysis (if standard analysis would not yield optimal results) maybe in our opinion a reasonable precaution, especially for samples for which no additional biological material is available. Saving HiSeq raw images is possible and relatively simple (it is described in Materials and Methods). These image files are large and must be transferred from the RTA computer in a timely manner, which should not be a problem in a well configured system. Unfortunately, there are currently no tools available for offline image analysis. For the HiSeq’s predecessor, Genome Analyzer II, both the manufacturer’s software (OLB) and third party software solutions for offline image analysis were available [7, 10]. Raw sequencing images generated by HiSeq are not only much bigger than those generated by Genome Analyzer II, but they are also logically partitioned in a very different way. Therefore, adapting the existing software to perform image analysis of raw HiSeq images with comparable quality with longer templates would require substantial effort.

As a proof of concept we analyzed the TIFF files offline (Table 5) to show that this is possible with the HiSeq platform by using an external device such as Drobo-S to store TIFF images (Materials and Methods). Offline image analysis was achieved by connecting Drobo-S to RTA workstation over eSATA port during sequencing, and thereafter it was connected to the offline analysis workstation. Images from top and bottom surfaces and each swath of the flowcell were analyzed separately. First, a shell script (freely available from the supporting webpage: http://instantseq.utmb.edu) was used to segment the 2048x160000 pixel images into tiles of smaller size and store them in a directory structure recognized by goat_pipeline (OLB 1.8.0) using tiffsplit utility. Next, goat_pipeline was invoked to analyze the images with Firecrest, and then Bustard was used to generate qseq files. Cycles used for template generation can be increased simply by editing the Makefile and adding --nd <number of cycles for template> and --first-detection-cycle <the first cycle from which template will be generated> where the image analysis executable is called.

**Table 5:** Offline image analysis of HiSeq images using goat_pipeline, which is designed for Genome Analyzer.

Tile Size (pixels)	Flowcell Surface	Template length	No. of Reads	% Barcoded
2048x2000	Bottom	19	∼300,000	41%
2048x5000	Top	6	∼200,000	17%

Columns: Image file size, flowcell surface from which image is obtained, number of cycles chosen for template generation in OLB software, number of reads obtained, percentage of reads with intact barcode. Offline image analysis was performed as proof-of-concept only and raw number of reads given should be understood as a rough estimate of the potential sequencing read yield from that method. The number of reads with the intact barcode (last column) can serve as a very conservative estimate of the number of usable reads.

The aforementioned offline analysis of the saved images, even with our proof-of-concept adaptation of the existing Genome Analyzer software, yielded promising results, which were substantially better than the results of online analysis of the same data with the standard template of length 4 (Table 1, top rows).

## Discussion

Here, we propose the Long Template Protocol (Materials and Methods), that allows low sequence diversity samples to be sequenced without quality problems and also improves the quality of sequencing of the normal samples and notably substantially reduces sample bleeding, i.e. incorrect assignment of the sequenced multiplexed samples. Our simple modification of the standard sequencing data analysis pipeline can correct or improve all these varied problems, because they are caused by the same issue: incorrect detection of the cluster boundaries on the flowcell during sequencing. The Long Template Protocol is the direct correction of the problem where it occurs.

Using Long Template Protocol, we successfully obtained very high-quality data from low or no diversity samples at most challenging conditions of normal cluster density (Table 1, Table 4). Specifically, in the examples presented in this paper, we used the Long Templates Protocol with the template lengths of 16 and 20. Theoretically, there is no drawback to generating templates based on more cycles, since it allows the software to better estimate cluster positions. In practice, this process is highly memory intensive. Therefore, the Long Template Protocol requires careful estimation of the memory requirements before attempting it. Such estimation can be performed by monitoring the ‘Memory Commit Size’ during sequencing in Windows Task Manager. This experiment should be done with the same cluster density as planned for the Long Template Protocol sequencing, as memory requirements may increase with the cluster density. As a rule of thumb, based on our experiments, templates of length 6 should never cause problems, templates up to length 12 can be tried cautiously, templates longer than 12 should be only attempted after upgrading memory of the RTA computer (Materials and Methods).

Even if choosing too long templates led to exceeding of the memory capacity of the RTA computer, there are several rescue strategies (lowering the number of computational threads, re-hybridizing the flowcell or off-line image analysis), that can be used, but they all lead either to lowered yields of sequencing or are very laborious. Therefore, for Long Template Protocol sequencing, we recommend choosing a very conservative template length to avoid complications, and to lower cluster density as an additional precaution. In case of limited non-diverse regions (such as barcodes), the highest improvement in quality will be seen for templates of length at least two plus the barcode length, since the cluster detection algorithm is using only the two best cycles (golden and silver) for cluster detection, and extending template generations by two cycles beyond the non-diverse region would allow the algorithm to pick the golden and silver cycle from fully diverse cycles. However, we have seen considerable improvement with even one of the template cycles being chosen from a fully diverse cycle. Moreover, during our many experiments, we also witnessed one example of the algorithm preferring a cycle from a non-diverse position over one from the fully diverse. Therefore, even if for technical reasons extending the template by two cycles beyond the initial non-diverse sequence length is not possible, extending template length should still increase data quality, although the result may not be as striking as those presented herein.

Using the Long Template Protocol should always improve data quality, even for samples without the low initial sequence diversity problem, although improvement will be more significant for the low diversity samples. Long Template Protocol substantially reduces “sample bleeding”, i.e. incorrect multiplexed sample assignments, because the root of the problem is the same as that for low quality data from low initial sequence diversity samples: errors in cluster calling. It has been reported in [10] that sample bleeding occurs orders of magnitude more often than previously thought and can lower the quality of data such as those used in studies of rare variants or cause incorrect diagnosis in multiplexed patient samples. Therefore, the approaches to improving sequencing data accuracy presented in this paper, especially use of the Long Template Protocol and lowering cluster density, can be used to achieve the level of sequencing accuracy necessary for clinical applications and for any other applications that require high sequencing accuracy.

For comprehensiveness, we also discuss other approaches to addressing the low-diversity problem and generally improving the data accuracy, although they are all more costly or laborious than implementing the Long Template Protocol (Table 6). The “low-diversity problem” is created at the software level, and can be addressed most simply and overcome effectively by software modifications, without the need to adjust experimental design or sequence unproductive samples such as PhiX. Moreover, some of the mentioned alternative approaches to rectify low diversity problem can be used only in specific situations. For example, re-designing barcodes would only be possible if the low diversity problem is caused by in-house barcodes, and not by inherent low diversity of the data (e.g. reduced representation sequencing). Moreover, even if redesigning barcodes is possible, it is laborious and costly (e.g. PCR primer optimization) and is not an option for sequencing existing samples.

**Table 6:** Comparison of different approaches to sequencing low-diversity data or maximizing sequencing accuracy.

Aproach	No.	Cost	Time	Limitations	After lib?	Rescue?	Recommendation
Long Template Protocol		$0	5 min	Template length limited by RAM memory	YES	YES	Most recommended (also reduces sample bleeding)
(Re-)design Diverse Barcodes	1	$$$- -$$$$	days/weeks	Applicable only to barcoded samples	NO	NO	Recommended only during barcode design
Lowering Cluster Density	2	$$$- -$$$$	5 min	Increases effective sequencing cost	YES	NO	Recommended (best use with Long Template)
Mixing with PhiX	3	$$$- -$$$$	5 min	Increases cost, causes sample bleeding	YES	NO	Not recommended (50% PhiX data, sample bleeding)
Rehybridize + Long Template	4	$$$	20 min	Requires kit purchase	YES	YES	Recommended for difficult to obtain samples
Offline Image Analysis	5	$0	days/weeks	Last resort strategy, labor intensive	YES	YES	Last resort (first try rehybridization)

Columns: approach name, approach number (for alternative approaches), extra cost ($$$—hundreds of dollars, $$$$—thousands), extra time needed, approach limitations, whether possible after library creation, whether feasible as a rescue strategy, summary recommendation. The cost of the Illumina rehybridization kit is currently ∼$400, cost of lowering cluster density or of mixing with PhiX was estimated from the resulting decreased number of reads from the sample of interest, cost and time necessary for developing new barcodes was estimated based on the need to design and optimize new PCR primers, etc.

The two other approaches to low diversity problem (mixing with diverse sample and lowering cluster diversity) have same goal—to increase distance between clusters from non-diverse samples on the flowcell. It can be achieved by two means: either by mixing a non-diverse sample with a diverse sample (Illumina recommends 50% PhiX spike-in) or by lowering cluster density by loading much less sample on the flowcell. Both these approaches have disadvantages. Mixing with PhiX is not recommended, since it leads to sequencing 50% unproductive samples, thus effectively halving sequencing yield and doubling the cost. Mixing a low diversity sample with a normal diversity sample of interest is better in that it does not lead to sequencing of unwanted samples, but it is also somewhat problematic. First of all, such experimental design can be difficult, especially for smaller groups, that may not be sequencing many and varied samples at the same time. Moreover, as we have shown, sample bleeding in the Illumina protocol is substantial, so even though normal sequencing yield is achieved from both samples, data quality is lowered by sample bleeding, at a level that can be unacceptable for high-accuracy applications. Therefore, if using this method, we recommend only to mix samples from distantly related organisms. Thus unavoidable sample bleeding will be less detrimental, since contaminant reads will be much less likely to map to the reference genome of the evolutionary distant sample, and therefore contamination will be substantially curtailed. We also caution sequencing facilities against multiplexing samples from different users—in such case, contamination will not only be occurring, but users will not know characteristics of the potential contamination (organism of origin or barcode), and therefore will not be able to identify reads most likely originating from sample bleeding, which may lead to detrimental and erroneous results in sequencing applications requiring high-accuracy. Lastly, from our repeated experiments with mixing our non-diverse (BLESS) samples with diverse ones, we observed that 40% of non-diverse sample was the upper limit that did not significantly alter data quality, but we set our target percentage of non-diverse sample to no more than 35% and simultaneously aimed for 75% of optimal cluster density to maximize the data quality. Depending on the protocol and equipment used in individual labs, level of control over the resulting cluster density and mixing ratio will vary. Therefore, since quality of data rapidly decreases both by exceeding optimal cluster density and allowable percentage of non-diverse data, the target values should be set rather conservatively, with margins depending on flowcell loading and sample mixing accuracy typically achieved.

Long Template Protocol can also be used as a rescue protocol; in such cases the same flowcell used in the unsuccessful sequencing run is sequenced again, this time using the Long Template Protocol instead of the standard Illumina protocol. We have shown that resequencing using the Long Template Protocol can strikingly improve the data quality: from several percent high-quality reads to a majority of reads being high-quality and mappable after Long Template Protocol sequencing (Table 2). This strategy can be especially useful if there is no remaining sample or sequencing libraries, except of those already loaded on the flowcell and sequenced. In principle, the same results—rescuing an unsuccessful sequencing run performed with the standard protocol—can be achieved by off-line analysis of the (previously saved) sequencing images using the Long Template Protocol. Due to the amount of effort required for this approach, however, we include it only as a proof-of-concept and do not currently recommend it.

At the time of submission of this paper, an upgrade of the Illumina primary data analysis software was released [9], which was intended to improve the low-diversity issue and related problems. Surprisingly software modifications did not address the issue directly, by allowing a flexible range and start cycle for template generation, but instead improved the phasing algorithm. To test the updated software, we compared two flowcells containing eight lanes of 20-40% low diversity samples multiplexed with normal diversity samples, one sequenced using the software before the upgrade, and one after the upgrade. Unfortunately, the problems with sequencing low diversity data remain in the upgraded software. The sample bleeding, which is a good overall indicator of a level of incorrect cluster calling and all issues related to low diversity of the samples, was reduced only by 1% (well within error margins). In contrast, we have shown that the Long Template Protocol can reduce sample bleeding by up to 80% (Table 6). Therefore, we still highly recommend Long Template Protocol to all interested in very high-quality sequencing data or working with low initial sequence diversity samples.

The purpose of this paper is two-fold: to present our indirect, but currently only possible, solution to the low-diversity problem and equally importantly to point out, in our opinion, undesirable trends in Illumina software development. Most of the previously available options have been currently eliminated from the Illumina software. While it is obviously important to streamline the process and provide a good default path through data analysis, in certain cases, such as ours, access to deprecated options will tremendously improve the results. The fact that Illumina is practically a monopoly and its software is closed-source is making this situation even more difficult. In spite of the closed-source nature of the software, we managed to adapt its parameters so it can perform much better for low-diversity data (Long Templates Protocol), but not all problems can be solved this way. Moreover, coinciding with the removal of the option to save images from the GUI, the promising third party software packages for image analysis ceased to be developed [7, 10]. Therefore, we explained above how images can still be saved. However, without more cooperative attitude of Illumina towards third-party scientific software developers it is unlikely efforts to develop alternative primary data analysis algorithms will be continued.

What can be done about this situation? Many research projects would benefit from Illumina making its software more flexible, at least returning to the stage it was for Genome Analyzer II, with additional options available e.g. only from command line. Ideal solution would be an open source option for offline primary data analysis. We believe that community pressure and involvement—to which awareness is a first step—would be very beneficial for future improvement of the Illumina software quality.

## Materials and Methods

### Sample preparation and sequencing

Initial BLESS samples (Table 1) were prepared as described in Crosetto et al. [5] and sequenced by imaGenes GmbH (Berlin, DE) using the Illumina HiSeq 2000 platform. For later experiments, 18 pM library from human gDNA per lane was loaded on a flowcell. Clusters were generated on flowcell v3 using an Illumina automatic cBot station and TruSeq PE Cluster kit v3. According to the manufacturer optimal cluster density for flowcell v1.5 and flowcell v3 is 400-450 K/mm^2^ and 750-850 K/mm^2^, respectively. Sequencing by synthesis was carried out using Illumina HiSeq 2000 system and TruSeq SBS Kit v3 chemistry. Rehybridization was done with Illumina cBot rehybridization kit.

Library preparation of NCS samples (BLESS labeled DNA from HeLA cells incubated with neocarzinostatin) was carried out using Illumina TruSeq DNA sample preparation kit v2, according to the standard protocol with some minor modifications. Briefly, 1000 ng of gDNA was end repaired into blunt ends without any prior fragmentation and a single ‘A’ nucleotide was added to the 3′ ends of the blunt fragments. Adenylated fragments were then ligated with Illumina indexed adapters and PCR amplified for selective enrichment without any previous size selection.

### Upgrading real time analysis (RTA) computer

For online data analysis, we used a Dell Precision 7500, one of the two computers (together with Dell 6900), routinely installed by Illumina as a Real Time Analysis computer for HiSeq 2000. The workstation has two 4 core Xeon X5667 3.06GHz CPU and 48GB RAM. There are two 3TB hard drives for local storage. We enhanced the local storage of the computer with a Drobo-S storage array with 5x3TB hard drives connected to the eSATA port. During our experiment all data including TIFF images and temporary files were stored on the Drobo-S array. After the first round of sequencing we upgraded the RAM to 192 GB. As an additional precaution against swapping we added a solid state drive, which is very fast compared to a typical hard drive (600MB/s read/write speed; 120,000 4K-random IOPS, whereas typical hard drives have a read/write rate of 100MB/s and 300-400 4K-random IOPS). We added 192GB RAM (only 128GB usable on Windows Vista), an OCZ Revodrive (for swap space) and a Drobo-S to store TIFF images. Two or more Drobo-S can be daisy chained to increase capacity. These modifications allowed us to successfully generate templates of length 16 and obtain high-accuracy sequenced data for the entire flowcell.

### Saving Illumina Sequencing Images

To save raw sequencing images to obtain data for offline image analysis, we modified the following XML files (HiSeq 2000): HCSconfiguration.xml, runParameters.xml and RTAconfig.xml. Within these files, the XML tags for enabling image file save, <SaveImages>, were modified.

Moreover, a file named “Save All Images.xml” was created in the PeriodicSaveRates folder with the following content:

 <?xml version=“1.0”?>
 <PeriodicSave Name=“Save All Images” Version=“1”>
 <SaveImages>true</SaveImages>
 <SaveThumbnails>true</SaveThumbnails>
 <UseZPositionNaming>false</UseZPositionNaming>
 <Surfaces>Both</Surfaces>
 <Swaths>Both</Swaths>
 <Channels>
 <Channel>A</Channel>
 <Channel>T</Channel>
 <Channel>C</Channel>
 <Channel>G</Channel>
 </Channels>
 </PeriodicSave>

To direct saved images to Drobo-S (optional) the following line was substituted in the config file, “HiSeqControlSoftware.Options.cfg”:

 <OutputFolder>*Folder with drive letter of Drobo*<OutputFolder>

If using HiSeq 2500, the same parameter in file “HiSeqControlSoftware.Options.cfg” should be changed. This file can be found in the directory Illumina/HiSeq Control Software/Configs.

### Generating templates of user-defined length (Long Template Protocol)

To generate user-defined templates using Illumina HiSeq Controlling Software, several XML configuration files need to be modified. The necessary changes can be implemented as a reversible patch. Additional guidelines can be obtained from the authors, if needed.

We start with description of changes for HiSeq 2000. The key modification for this protocol is to increase the number of cycles used for template generation (it is currently impossible not to include initial low-diversity cycles in template generation, so to include later diverse cycles template length must be increased). To this end, the default value “4” of the XML tag <TemplateCycleCount> was changed to the desired value (target template length), of 20, as follows:

 <TemplateCycleCount>**20**</TemplateCycleCount>

Saving raw sequencing images is an optional part of the Long Template Protocol and is enabled by modifying the “HiSeqControlSoftware.Options.cfg” as described here. When performing the Long Template Protocol with image saving option, we began by installing a copy of Illumina HCS and RTA software on the Drobo-S storage device and then followed steps outlined in section Saving Illumina Sequencing Images above. To change template length using HiSeq 2500, the parameter <TemplateCycleCount> in file “HiSeqControlSoftware.Options.cfg” should be changed. This file can be found in the directory Illumina/HiSeq Control Software/Configs.

### Reducing number of execution threads

This is a rescue strategy that would reduce memory requirements for template computation, but would also result in partial loss of data. As such, it should only be used as a last resort, for example if memory requirements were accidentally exceeded by setting templates so long that the template generation cannot be finished in an acceptable time. To reduce number of execution threads, the “NumAnalysisThreads” parameter in the config file, “HiSeqControlSoftware.Options.cfg”, must be changed from the default value of 8 to a desired lower value, for example 4:

 <NumAnalysisThreads>**4**</NumAnalysisThreads >

### Barcode Trimming, Filtering and Read Mapping

FASTQ files were generated using Illumina OLB 1.93 and CASAVA 1.8 tools. BLESS reads have one of the two 11nt long barcodes, either TCGAGGTAGTA or TCGAGACGACG. This property was used to filter out such reads from yeast control data. Reads were pre-processed using our in-house *Instant Sequencing* software, only reads containing at least 34 consecutive bases with Phred scores above 20 were retained and referred to as high-quality reads. Bowtie2 [16] was used to align reads to reference genomes: human (hg19), S. cerevisiae (May 09) and PhiX 174, respectively.

### Calculation of sample bleeding

Sample bleeding was estimated as follows: First, “expected” sample bleeding from human to yeast and and human to PhiX samples was determined. To this end, we sequenced two 100% human samples, for which sequencing libraries were prepared in a manner excluding any chance of cross-contamination. Before mapping, all reads underwent our strict filtering procedure and only reads with high quality scores for every base called were retained for mapping. From these samples, the “expected” sample bleeding was computed; i.e. the number of reads originating from 100% human sample that, due to sequencing errors, would not map without mismatches to the human genome, but would map, again without mismatches, to yeast or PhiX genome, divided by the total number of reads remaining after read quality filtering, described above. This ratio is the expected false positive ratio and based on calculation in these two independent samples it is 8.5*e* ^−7^ ± 0.4*e* ^−7^ for expected “false positive” mapping of reads originating from a human sample to the yeast genome. Since we did not find in our human samples any reads not mapping to the human genome, but mapping without mismatches to the PhiX174 genome, as an estimate of the false positive mapping to the PhiX174 genome we assumed a ratio smaller than an inverse of the number of the reads in each of the respective samples.
