# Correction: Strategies for achieving high sequencing accuracy for low diversity samples and avoiding sample bleeding using illumina platform

**DOI:** 10.1371/journal.pone.0227431

**Published:** 2020-01-02

**Authors:** The PLOS ONE Staff

The images for Figs 1 and 2 are incorrectly switched. The image that appears as Fig 1 should be Fig 2, and the image that appears as Fig 2 should be Fig 1. The figure captions appear in the correct order. Please view Figs 1 and 2 with the correct captions here.

**Fig 1:**
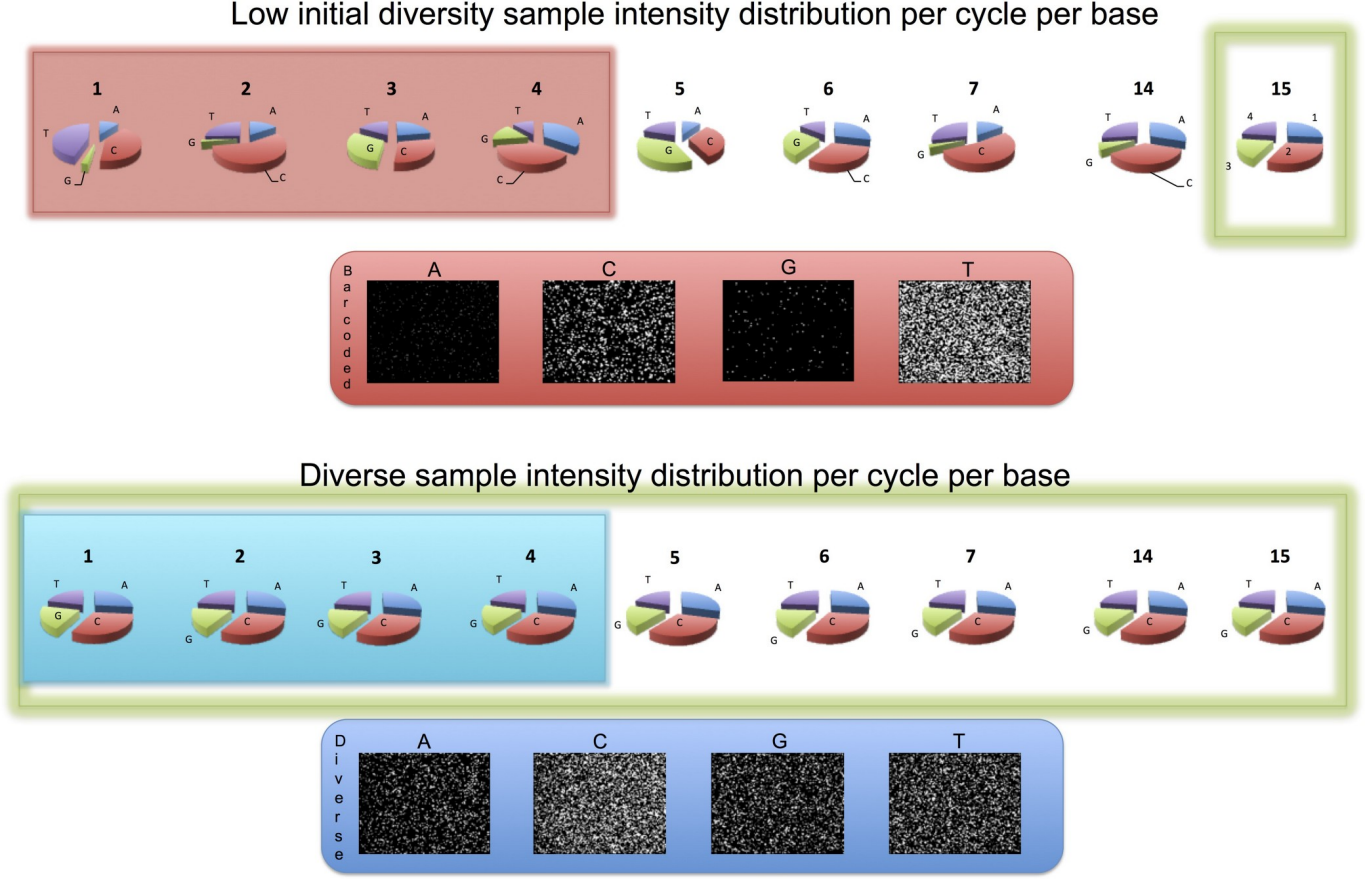
Example of differences between base frequencies and raw images from sequencing a low initial sequence diversity sample and a diverse sample. The low initial sequence diversity sample is highlighted in red (top panel) and a diverse sample is highlighted in blue (bottom panel). Per cycle intensity (which correlates with base frequency) pie charts are shown at the top for each sample. The low diversity sample (top) has an 11bp 5′ -barcode, that causes different base frequencies than in the diverse human sample (bottom). For each sample, the bottom panel shows raw sequencing images from the first cycle of sequencing. The imbalanced frequency distribution in the top panel (barcoded, low diversity sample, highlighted in red) is due to over-representation of the base ‘T’, the first base in the barcode. There is also some signal from base ‘C’ due to linker sequencing.

**Fig 2:**
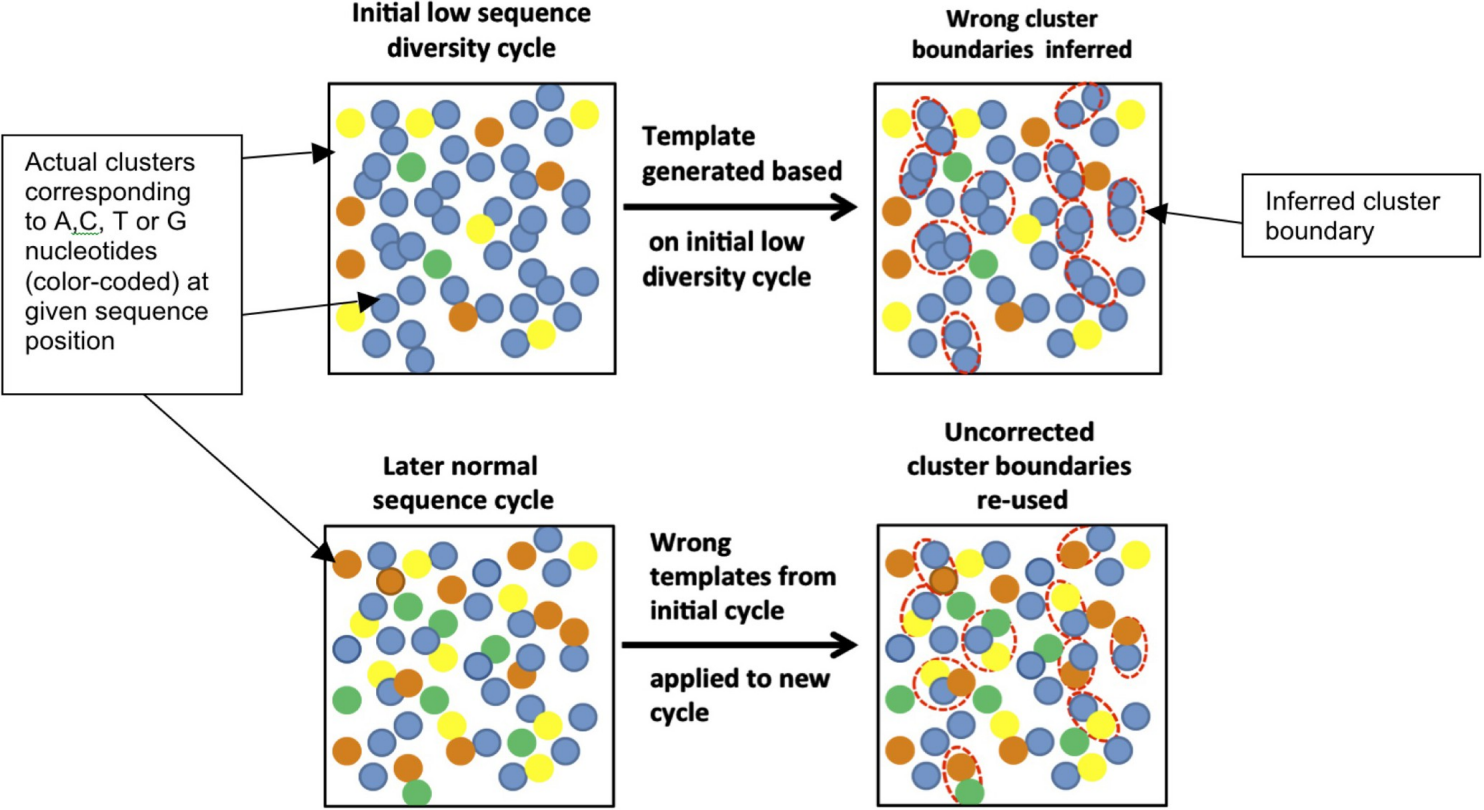
Lack of sequence diversity in first four positions causes errors in base calling in later cycles. Template used for cluster calling is created using only data from the first four cycles (top). Once the template is generated, it is never corrected (bottom). Templates created based on low-sequence diversity cycles tend to be of poor quality (bottom right) and result in poor cluster detection for later, normal diversity cycles as well, thus globally compromising sequencing quality.
